# Gastric Hyperacidity

**Published:** 1903-05-09

**Authors:** 


### GASTRIC HYPERACIDITY.

There are few affections of which the accounts given in the ordinary text-books are more confusing, or whose nomenclature is more vague than the different forms of dyspepsia. In an address, given before the Edinburgh Medico-Chirurgical Society, and published in the *British Medical Journal*, Dr. William Russell called attention to some important facts in connection with that form of indigestion which is associated with excessive secretion of hydrochloric acid. In his opinion this increase of hydrochloric acid is a more frequent cause of dyspepsia, and ultimately of gastric dilation, than is usually recognised. In many cases classed as neurotic, hyperchlorhydria will be found. The symptoms in this condition are characteristic, and consist mainly of epigastric pain or discomfort, coming on from one to three hours after a meal, and relieved when more food is taken. The pain may be of all grades of severity. Other symptoms are flatulent distension, gaseous and acid eructations and heartburn. The eructation of gas or of a small quantity of acid material usually gives temporary relief, often very marked and out of all proportion, apparently, to the amount brought up. There may be great physical and mental misery, langour, weariness, and inability to apply mind or body to any work, coming on or attaining their worst when other symptoms are at their height. In the process of digestion as it normally occurs the conversion of insoluble starch into soluble starch and finally into maltose is effected by the ptyalin of the saliva, the process being continued in the stomach until by the secretion of hydrochloric acid the gastric contents acquire an acidity of 0·003 per cent. or thereabouts when the digestion of starch ceases. The digestion of proteids is accelerated by the increasing amount of hydrochloric acid. In normal cases no more gastric juice is secreted than can be engaged by the amount of proteid which the stomach contains. If on the other hand the secretion of hydrochloric acid continues beyond this point discomfort will arise. The proteid elements of the food are digested with exceptional rapidity, and it might be thought that the other food substances, such as starch, would readily pass out of the stomach with the dissolved proteids; but this is not the case, for a considerable portion of unaltered starch remains in the stomach. The effect of this retained and undigested starch is to keep up the gastric secretion without providing any substance with which the hydrochloric acid can combine. Not only starch, but the fat of milk, plain or peptonised, has been found by Dr. Russell retained in this way. When the stomach contents are examined at this period they often measure only from one to a few ounces and consist of a very acid material, mainly fluid, containing starch, fat, and shreds of undigested material according to the food that has been taken. Starch is the main constituent. Once the residuum in the stomach becomes hyperacid, its retention is explained by the fact that the hyperacidity keeps the pyloric sphincter closed. As to the etiology, Dr. Russell is of opinion that certain individuals are prone to secrete over-much hydrochloric acid. The effects upon the stomach wall are catarrh and, later, atomic dilatation. With regard to treatment, an alkali taken some time before food neutralises any acid residuum and facilitates the transformation of the starchy elements of the next meal. In other instances relief is obtained by the administration of an alkali as soon as discomfort begins. Concerning diet, the most important restrictions are with respect to carbohydrates, which must be limited in quantity and must consist of the most soluble kinds. In the worst cases a purely proteid diet should be used for a short period. In all cases the amount of food taken must be duly regulated.

